# Sternum drop during trip recovery differs between the laboratory and real world – An exploratory pilot study

**DOI:** 10.1371/journal.pone.0328621

**Published:** 2025-07-17

**Authors:** Youngjae Lee, Neil B. Alexander, Christopher T. Franck, Michael L. Madigan

**Affiliations:** 1 Grado Department of Industrial and Systems Engineering, Virginia Tech, Blacksburg, Virginia, United States of America; 2 Department of Internal Medicine, Division of Geriatric and Palliative Medicine, University of Michigan, Ann Arbor, Michigan, United States of America; 3 Veterans Affairs Ann Arbor Health Care System Geriatric Research Education and Clinical Center, Ann Arbor, Michigan, United States of America; 4 Department of Statistics, Virginia Tech, Blacksburg, Virginia, United States of America; University of Giessen: Justus-Liebig-Universitat Giessen, GERMANY

## Abstract

The goal of this exploratory study was to compare sternum drop—the decrease in sternum height during an attempt to recover balance after tripping—between lab-induced trips and naturally occurring real-world trips. Twenty community-dwelling adults 71.8 (4.6) years old used three inertial measurement units (IMUs) and a wrist-worn voice recorder daily for three weeks to capture sternum drop during any naturally-occurring real-world trips. Participants then completed a single laboratory testing session during which they were intentionally exposed to two lab-induced trips while wearing the same IMUs to also evaluate sternum drop. All real-world trips resulted in recoveries while only 12 of the 22 lab-induced trips resulted in recoveries (the remaining 10 were falls). When including all lab-induced trips, sternum drop after real-world trips was 8.8 cm smaller (*p* < 0.001), exhibited less variance (*p* < 0.001), and was not associated with lab-induced trips (*R*^*2*^ = 0.005; *p* = 0.757). When only including lab-induced trips that resulted in recoveries, sternum drop after real-world trips did not differ from the lab (*p* = 0.163), exhibited less variance (*p* < 0.001) and was not associated with lab-induced trips (*R*^*2*^ = 0.006; *p* = 0.766). These results were likely dependent upon 1) our lab protocol that required participants to walk at a gait speed that was likely faster than typical gait speed in the real-world, and 2) the aggressive lab tripping obstacle height of 8.6 cm was likely taller than at least some real-world trips. While reducing gait speed and obstacle height in future laboratory studies may improve agreement with real-world trips, this would lower the physical demands during recovery and may not be as effective at revealing factors contributing to falls. Nevertheless, additional research appears warranted to clarify the linkage between lab and real-world trips. To our knowledge, this is the first study comparing tripping kinematics between the lab and real world.

## Introduction

Falls are the leading cause of fatal and non-fatal injuries among older adults [1] with annual medical costs of $50 billion [2]. An estimated 29–53% of falls among community-dwelling older adults result from tripping [3–5]. As such, numerous laboratory studies have induced trips in a cohort of older adults to explore the biomechanical mechanisms underlying trip-induced falls [6–8], to identify factors affecting trip recovery [9–11], and to evaluate the efficacy of interventions aiming to reduce the prevalence of trip-induced falls [12–16]. While these studies have yielded a wealth of knowledge, we are not aware of any studies that have compared the kinematics of lab-induced trips to those that naturally occur in the real world. Any differences between these two settings may influence the ability to generalize laboratory findings to the real world.

Successfully recovering balance after tripping has three kinematic requisites: limit trunk flexion angle, maintain adequate hip height to enable repeated stepping, and complete recovery steps to extend the base of support anterior to the center of mass [6–8,14,17–19]. The inability to achieve any of these three requisites likely results in a fall. Thus, tripping kinematics are commonly evaluated using measures related to these three requisites. Sternum drop, or the decrease in sternum height during an attempt to recover balance after tripping, is geometrically dependent upon trunk flexion angle and hip height [20], and may be a useful trip recovery measure given its dependence upon two of the three requisites for successful trip recovery. Indeed, sternum drop has been validated by showing that 1) it is larger after lab-induced trips resulting in a fall into a safety harness compared to those resulting in a successful balance recovery, and 2) it is highly correlated with limiting trunk flexion angle and maintaining adequate hip height [20]. It also has the benefit of being amenable to capture using inertial measurement units, which is strongly correlated with sternum drop from a gold-standard optoelectronic motion capture system [20] and thus appear viable as a trip recovery measure for outside the laboratory setting.

The goal of this exploratory study was to compare sternum drop between lab-induced trips and naturally occurring real-world trips among community-dwelling older adults. Three hypotheses were posed. First, we hypothesized sternum drop would be smaller after real-world trips than after lab trips. This was based upon evidence that the vast majority of real-world trips result in recoveries [21,22] while a higher percentage of lab trips result in falls. This implies real-world trips are, on average, less severe than lab trips, and as such should exhibit smaller sternum drop. Second, we hypothesized sternum drop would exhibit greater variance after real-world trips than after lab-induced trips. This was based upon anecdotal evidence that real-world tripping scenarios are more varied and diverse than those in controlled laboratory studies due to wider-ranging environmental factors (e.g., ground/floor/trip obstacle material properties and geometry) and behavioral factors (e.g., gait speed, dual-task participation, attention, and awareness). Third, we hypothesized sternum drop after real-world trips would be associated with sternum drop after lab-induced trips. This was based on the expectation that balance recovery ability is a personal skill that would have similar influence on tripping kinematics after real-world and lab-induced trips. The results of this study will begin to clarify the relationship between lab-induced trips and real-world trips. It may also inform the design of future laboratory tripping studies if there is a desire to mimic real-world trips in the lab.

## Materials and methods

Participants included 20 community-dwelling older adults (8 M and 12 F) from a larger study investigating perturbation-based balance training (PBT) [23]. These 20 participants were chosen because they were allocated to either a treadmill-based PBT intervention (*n* = 10) [14,24] or a control group receiving no intervention (*n* = 10). Participants had a mean (SD) age of 71.8 (4.6) years, body height of 1.70 (0.11) m, body mass of 80.8 (15.9) kg, and unipedal stance time of 16.9 (12.9) sec (seven participants were below 5 sec indicating high fall risk [25]). Participants were recruited from the university and local community via email listservs, flyers, word-of-mouth, and visits to local community organizations. Eligibility required: age 65–80 years old; no current back, leg, or foot pain that interfered with standing or walking; no lower limb amputation; no hospitalizations in the last six months; Montreal Cognitive Assessment score ≥ 19 [26]; bone mineral density of the lumbar spine or proximal femur *t* > −2.5 (Lunar iDXA, GE Healthcare, Chicago, IL); no history of hip or vertebral fracture; no dependency on assistive device to walk; no regular engagement in exercise to improve balance; and willingness to wear a voice recorder and three IMUs for three weeks (see Methods below). Participant recruitment was from October 6, 2022 to June 26, 2023, and data collection from the last participant was completed on August 3, 2023. The study was approved by the Virginia Tech Institutional Review Board (VT IRB# 21-1072), and all participants provided written consent prior to participation.

A repeated-measures experimental design was used. Although not the primary focus here, participants first completed three weeks of twice weekly PBT or no intervention as a control. Afterward, and the primary focus here, participants wore three IMUs and a wrist-worn voice recorder daily for three weeks to capture real-world loss of balance (LOB) kinematics and context. Participants then completed a single testing session during which they were exposed to two lab-induced trips while wearing the same three IMUs. Only data from the three weeks of real-world loss of balance capture and the post-intervention testing session were analyzed here.

Participants visited the laboratory to be issued the devices and to receive instructions and training on their use. Participants were asked to don the IMUs (Opal, APDM, Inc., Portland, OR) and voice recorder (Mini Wrist Band Voice Activated Recorder, SpyCentre Security, Plano, TX) each day when they were ready to start their daily activities in the morning, wear the devices throughout the day (other than while bathing or activities that could result in them getting wet), and remove the devices when they finished their daily activities in the evening. This schedule was chosen because IMU battery life was approximately 12 hours and required overnight charging. The times the devices were worn by participants ranged from 6 am to 10 pm based on participant preference. One IMU was worn on sternum using the manufacturer’s chest strap harness, and the other two were worn on the dorsum of the feet using the lab-provided shoe pouches that were secured to the shoelaces. IMU data were sampled at 128 Hz and continuously logged onboard the IMUs from the moment they were unplugged from the charger in the morning to the moment they were plugged back into the charger in the evening. The voice recorder was worn on the wrist and used to document the context of any real-world LOB immediately after it was experienced or as soon after as possible. A LOB was defined as “a sudden, unexpected change in body position that requires us to do something to regain our balance or else we will fall.” Examples of LOB context included answers to questions when, where, and why the LOB occurred, and what the participant was doing at the time of the LOB. Details of the voice recordings are reported elsewhere [27]. Voice recordings also provided a time stamp of the LOB that aided in finding LOBs within IMU data. The investigators contacted participants via phone call or text at least once during each of the three weeks to address any questions or issues. Participants also visited our laboratory at the end of each week to allow the investigators to download all data from the devices and address any issues or questions from the participants.

After the three weeks of real-world LOB capture, participants completed a single post-intervention testing session during which they were exposed to two lab-induced trips while walking overground using methods described elsewhere [24,28]. Briefly, participants wore the same three IMUs that they wore during the real-world LOB capture and were asked to complete multiple walking trials on a 12-meter level walkway at a purposeful speed (i.e., as if they were going somewhere) while looking straight ahead. To minimize any preparatory adaptations to the trip perturbation, participants were informed that they may or may not be exposed to one or more trips or slips while walking, and if so, to simply react naturally and then continue walking. After completing a minimum of 10 walking trials, a trip obstacle integrated into the walkway was actuated by the investigators at the start of the stance phase of the non-dominant foot to abruptly raise to a height of 8.6 cm to induce a trip to the dominant foot during the mid-to-late portion of its ensuing swing phase. Attempts were then made to induce a slip (reported elsewhere [29]) and then one additional trip, with a minimum of three walking trials between perturbations. Participants wore standardized footwear (Model 411 walking shoes, New Balance Athletics, Inc.) and a safety harness attached to an overhead track along the walkway to prevent a fall to the floor in the event of an unsuccessful balance recovery.

Force applied to the safety harness was sampled at 1280 Hz using a uniaxial load cell (Cooper Instruments, Warrenton, VA) and low-pass filtered at 40 Hz (fourth-order, zero-phase-lag, Butterworth filter). If the integrated harness force from trip onset to one second after touchdown of the first recovery step did not exceed 20% body weight * second, a trip was classified as a recovery. Otherwise, a trip was classified as a fall (i.e., receiving substantial support from the safety harness). A missed trip occurred if the leading edge of the swing foot did not contact the trip obstacle during mid-to-late swing phase because the trip obstacle was triggered too late by the investigators. Subsequent data processing was performed using custom code in MATLAB R2021a (The MathWorks Inc., Natick, MA).

**Fig 1:**
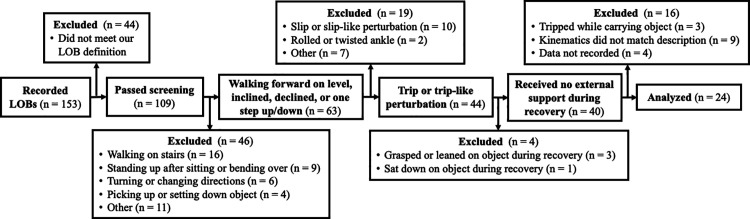
Flow diagram showing screening process for identifying real-world trip-induced LOBs that were corroborated by the IMU data.

During the three-weeks of real-world LOB capture, participants wore the IMUs and voice recorder for a mean (SD) of 10.9 (0.6) hours per day and for 20.7 (0.5) days. Twenty-two participants wore the devices for the full 21 days, seven used it for 20 days (returned the devices one day early to accommodate scheduling constraints), and one used it for 19 days (did not wear the devices for two days while sedentary and recovering from illness) [27]. A total of 153 LOBs were reported from 18 of the 20 participants (two participants did not report any). LOBs were then identified within the IMU data using the time stamps of the voice recordings and/or a description of exact time that the LOB occurred. These LOBs were then screened to limit subsequent analyses to those that were described as a trip or trip-like perturbations (e.g., “caught foot on”, “stubbed foot”). Nine of these events were excluded from further analysis because they did not have IMU data that corroborated these descriptions [21] in that the kinematic data showed no appreciable changes in trunk and/or stepping pattern so that we could not identify trip onset or touchdown of the first recovery step. This screening process yielded a total of 24 real-world trips from 14 of the 20 total participants who were walking forward (not on stairs) without carrying anything, received no external support, and showed recognizable tripping kinematics from all three IMUs (Fig 1). Two of the 14 participants experienced three and five trips or trip-like perturbations, respectively, while the remaining 12 participants experienced one or two trips or trip-like perturbations.

Tripping kinematics were characterized using sternum drop—defined to be the decrease in sternum IMU height from trip onset to touchdown of the first recovery step. Successful balance recovery after tripping requires limiting any trunk flexion and drop in hip height [7,8]. Because sternum drop would increase as trunk flexion angle increases and as hip height decreases [20], an increase in sternum drop toward positive infinity is indicative of poorer trip recovery performance. Moreover, sternum drop, as captured here using an IMU worn on the sternum, exhibits high (*r *> 0.90) correlation with sternum drop, trunk angle at touchdown, and hip height at touchdown when measured using an optoelectronic motion capture system, and differs between trip-induced falls and recoveries [20]. To facilitate the measurement of sternum drop during the three weeks of real-world LOB capture, participants were asked to perform a series of calibration movements upon first donning the IMUs in the morning and then again at lunchtime or whenever they changed clothes or shoes. These calibration movements were also performed prior to the first trip during the testing session in our laboratory. The IMU data collected during these movements were used with a principal component analysis to align the IMU local frame with the anatomical planes [30]. Sternum drop was determined by double-integrating the vertical acceleration of the sternum IMU from trip onset to touchdown in a coordinate frame and direction that stayed aligned with gravity throughout trip recovery. To minimize drift, the average vertical acceleration measured by this IMU when the participant was stationary prior to each real-world or lab-induced trip was subtracted before the double integration. A time when the participant was stationary prior to each real-world trip was found by scrolling backward in time within the feet and sternum IMU acceleration data to find a time point when the participant appeared to be standing stationary based upon visual inspection of these data. The time when the participant was stationary prior to each lab-induced trip was several seconds of standing stationary prior to each trial. We also note that some trips exhibited a negative sternum drop that indicated an increase in height from trip onset to touchdown of the first recovery step. This may have occurred due to relative movement between the shoulder harness holding the sternum IMU and the sternum itself during tripping, or during the time interval over which the IMU acceleration data was integrated up until the trip.

Several statistical analyses were performed. Our first hypothesis was that sternum drop would be smaller after real-world trips than after lab trips. To address this hypothesis, we investigated differences in sternum drop between the real world and laboratory using a mixed-model analysis of variance (ANOVA) with factors of setting (real world or laboratory), training group (PBT or control), and participant as a random variable to account for repeated trips. We also used a one-way repeated-measures ANOVA to investigate differences between the two lab-induced trips. Our second hypothesis was that sternum drop would exhibit greater variance after real-world trips than after lab-induced trips. To address this hypothesis, we investigated differences in the variance of sternum drop between the real world and laboratory using Levene’s test on the residuals of the mixed-model ANOVA. We used the residuals of the mixed-model ANOVA for this analysis because the residuals captured the remaining variability in these data after accounting for setting, training group, and the repeated trips. Our third hypothesis was that sternum drop after real-world trips would be associated with sternum drop after lab-induced trips. To address this hypothesis, we used the coefficient of determination (*R*^2^) to investigate the association between each real-world trip(s) and the mean of the two lab-induced trips for each participant. Because all real-world trips resulted in recoveries while only some lab-induced trips resulted in recoveries (see Results), these three analyses were performed twice. The first set of these analyses included all real-world and lab-induced trips and provided a wholistic analysis of the data. The second set of analyses included all real-world trips and only lab-induced trips that resulted in recoveries. This latter set of analyses was included in the event that including falls in the lab induced some bias within the data. Both analyses were viewed as important to gain additional perspective of the data. Statistical model-based means and 95% confidence intervals (CI) of sternum drop were reported. Statistical analyses were performed using JMP Pro 16 (SAS Institute, Inc., Cary, NC) with a significance level of 0.05.

## Results

All 24 real-world trips resulted in successful balance recoveries. Among the 14 participants (*n* = 7 each in PBT and control groups) who reported at least one real-world trip, their 28 lab-induced trips resulted in 12 recoveries, 14 falls, and two missed trips. Lab-induced trips were excluded from analyses if they were missed (*n* = 2), had corrupted harness force data (*n* = 1), or involved >30% body weight applied to the safety harness before touchdown of the first recovery step that may have artificially influenced tripping kinematics (three). The analysis then proceeded with sternum drop data from 22 laboratory trips (10 of which were falls) and 24 real-world trips.

**Fig 2:**
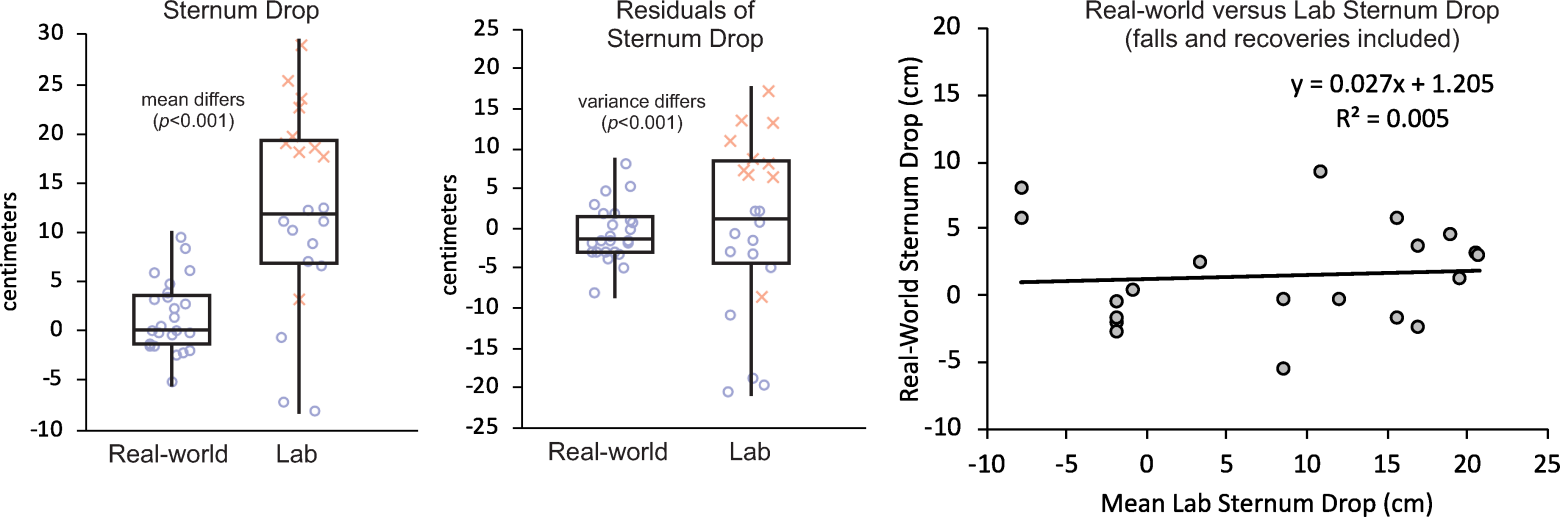
(Left) Box and whisker plot comparing sternum drop and (Middle) variance of residuals of sternum drop after a mixed-model ANOVA between real-world trips and lab-induced trips. Boxes illustrate median, first quartile, and third quartile. Whiskers illustrate max and min values. Individual values are shown with blue circles indicating recoveries and red crosses indicating falls. (Right) Scatter plot and linear fit of real-world sternum drop as a function of mean laboratory sternum drop. Note that the mean of the two lab-induced trips from each participant was used as the *x*-coordinate in this plot, so multiple real-world trips from a participant were assigned the same *x*-coordinate. Note that only 21 real-world trips were included in this analysis because one participant who experienced three real-world trips had both lab-induced trips excluded because >30% body weight applied to the safety harness before touchdown of the first recovery step.

Results differed depending upon whether falls after lab-induced trips were included. When including all falls and recoveries after lab-induced trips (Fig 2), sternum drop was a mean of 8.8 cm smaller in the real world (2.2 cm; CI: 1.6–6.0 cm) than in the laboratory (11.0 cm; CI: 7.1–14.8 cm; *p* < 0.001). Sternum drop did not differ between training groups (*p* = 0.626) or between the two lab-induced trips (*p* = 0.759). The variance of residuals of sternum drop after using a mixed-model ANOVA to account for the effects of setting, training group, and repeated measurements was lower in the real world (*σ*^2^ = 12.6 cm^2^) than in the laboratory (*σ*^2^ = 120.2 cm^2^; *p* < 0.001). Sternum drop in the real world was not associated with the mean of each participant’s two lab-induced trips (*R*^2^ = 0.005; *p* = 0.757).

**Fig 3:**
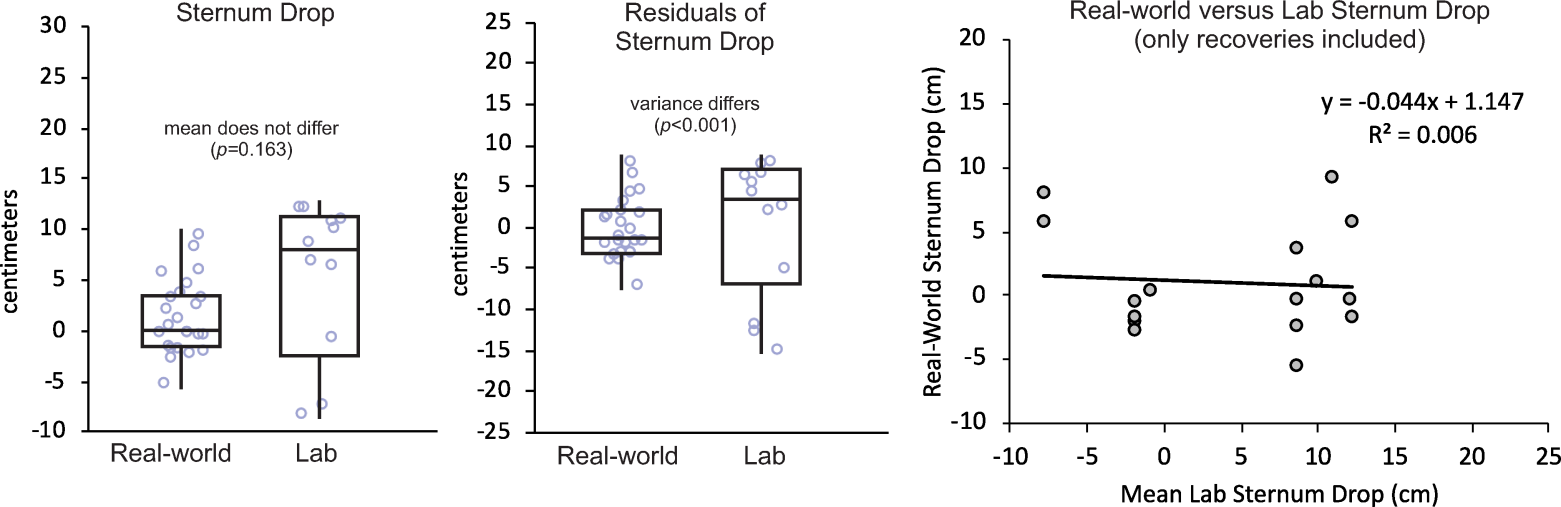
(Left) Box and whisker plot comparing sternum drop and (Middle) variance of sternum drop residuals after mixed-model ANOVA between real-world trips and lab-induced trips. While Fig 2 illustrates results when both falls and recoveries were included in the analyses, this figure illustrates results when only recoveries were included. Boxes illustrate median, first quartile, and third quartile. Whiskers illustrate max and min values. Individual values are shown with blue circles. (Right) Scatter plot and linear fit of real-world sternum drop as a function of mean laboratory sternum drop. Note that the mean of the two lab-induced trips from each participant was used as the *x*-coordinate in this plot, so multiple real-world trips from a participant were assigned the same *x*-coordinate.

When including only recoveries after lab-induced trips (Fig 3), sternum drop in the real world (1.5 cm; CI: −1.1–4.2 cm) did not differ from the laboratory (4.4 cm; CI: 0.9–8.0 cm; *p* < 0.163). Sternum drop did not differ between training groups (*p* = 0.767) or between the two lab-induced trips (*p* = 0.419). The variance of residuals of sternum drop after using a mixed-model ANOVA to account for the effects of setting, training group, and repeated measurements was lower in the real world (*σ*^2^ = 13.1 cm^2^) than in the laboratory (*σ*^2^ = 74.2 cm^2^; *p* < 0.001). Sternum drop in the real world was not associated with the mean of each participant’s two lab-induced trips (*R*^2^ < 0.01; *p* = 0.766). The data reported here is available as a supplementary file on the publisher’s website.

## Discussion

The goal of this exploratory study was to compare sternum drop between lab-induced trips and naturally occurring real-world trips among community-dwelling older adults. We acknowledge that this study is based on a small sample size and thus may have limited generalizability. However, to our knowledge, this is the first study comparing tripping kinematics between the lab and real world, thus attempting to clarify the relationship between tripping kinematics in these two settings. While all 24 real-world trips resulted in recoveries, only 12 of 22 (55%) lab-induced trips resulted in recoveries with the remaining 10 trips resulting in a fall. Knowing that sternum drop tends to be larger after falls than recoveries [20], it was perhaps not unexpected that support for our first hypothesis was dependent upon whether all lab-induced trips were included in the analysis or just recoveries were included. Support (or lack thereof) for our second and third hypotheses did not differ whether lab-induced falls were included in the analyses.

Our first hypothesis, that sternum drop would be smaller after real-world trips than after lab trips, was partially supported. When comparing real-world trips (all of which were recoveries) to lab-induced trips resulting in both falls and recoveries, sternum drop was smaller in the real world than in the lab. When only comparing real-world trips to lab-induced trips resulting in recoveries, sternum drop did not differ between settings. This suggests the larger sternum drop values when the participants fell in the lab (Fig 2) were responsible for the differences between settings. This can likely be explained by the real-world trips being less severe and requiring lower physical demands to prevent a fall than lab-induced trips. There are at least two potential reasons for this. First, the height of the trip obstacle in the laboratory was 8.6 cm while many real-world trips were caused by lower height obstacles [27]. For example, several voice recordings described real-world trips where a shoe sole scuffed on a flat floor surface during swing and stopped its forward motion – as if the shoe had impacted a rigid obstacle. In this situation with a lower height obstacle (or no obstacle) in the real world, balance recovery would be less extreme or challenging because the recovery step would not need to be elevated as much or at all to clear the obstacle (all other factors considered equal) [17,31]. For comparison, other laboratory tripping studies have employed obstacle heights over a range from 5 cm [8,14,31] up to 15 cm [7,32].

The second potential reason for considering real-world trips to be less severe and requiring lower physical demands than lab-induced trips was that walking speed in the laboratory was 1.55 (0.17) m/s and likely faster than the walking speed during at least some (if not many) of the real-world trips. Participants in the laboratory were asked to walk at a purposeful speed (i.e., as if they were going somewhere) to increase the physical demands of successful balance recovery to sufficiently challenge the balance control system, and thus better reveal any decrements in balance recovery ability. Faster gait speeds would result in faster or more extreme tripping kinematics when the forward translational momentum of the body prior to trip onset is converted to angular momentum around the trip obstacle at trip onset [33]. Although we did not measure gait speed in the real world, it is likely that it was slower than in the laboratory for at least some real-world trips due to participants likely walking at a more comfortable speed than in the laboratory, and because some real-world trips occurred near gait initiation when walking speed may not have increased fully to the desired speed yet [27]. Other possible reasons for real-world trips being less severe than lab-induced trips may exist.

Our second hypothesis, that sternum drop would exhibit greater variance after real-world trips than after lab-induced trips, was not supported. In fact, our data supported an opposite effect with smaller variance after real-world trips than lab-induced trips. Anecdotally, we expected environmental factors (e.g., ground/floor/trip obstacle material properties and geometry) and behavioral factors (e.g., gait speed, dual-task participation, attention, and awareness) to be more varied in the real world than in the laboratory. In retrospect, two of these factors that may have contributed to our findings were the likely lower trip obstacle height and slower gait speed for at least some real-world trips when compared to the lab-induced trips. Both of these, as noted above in the previous paragraph, would generally result in less extreme or a smaller mean sternum drop. A smaller mean would likely reduce the range of sternum drop values across all real-world trips and thus result in less variance. It should be noted that due to the repeated-measures experimental design employed here, any intrinsic risk factors within some or all participants would be balanced across both settings and thus not a threat to internal validity.

Our third hypothesis, that sternum drop after real-world trips would be associated with sternum drop after lab-induced trips, was not supported. Greater association between real-world and lab-induced trips may require more closely matching environmental (e.g., obstacle height) and behavioral (e.g., gait speed) factors between the two settings. Yet, even during laboratory studies that aim to control these factors, tripping kinematics across multiple overground or simulated trips can be inconsistent [34] given their dependence on whole body kinematics at trip onset (i.e., the initial conditions), complex and potentially wide-ranging response dynamics, and central set [35] among other factors. As such, the lack of association found here is perhaps not unexpected. An individual’s maximum capacity to recover balance when exposed to a simulated or naturally occurring trip is an intrinsic fall risk factor that, at least conceptually, can be reliably evaluated [36–38]. However, balance recovery after many real-world and lab-induced trips involve physical demands that likely do not approach this capacity, and as a result do not necessarily reflect an individual’s balance recovery capacity.

Several limitations of this study warrant discussion. First, as described above, our results are likely dependent to some degree upon our laboratory methodology including our choice to use a “purposeful” or slightly hurried gait speed and the 8.6 cm height of the trip obstacle. Future studies are needed to better understand these potential differences between settings. Second, the lab-induced trips involved wearing a safety harness while the real-world trips did not. To minimize any such influence, we excluded lab-induced trips during which > 30% body weight (a criterion used to determine substantial support [39,40]) was applied to the harness before touchdown of the first recovery step over the trip obstacle. Moreover, the sense of security afforded by the harness may have induced some participants to not provide their best effort to recover balance and thus resulted in more extreme tripping kinematics while the lack of safety harness in the real world would not have the same effect. If this occurred, it would likely have increased the variance in tripping kinematics in the laboratory. Third, we limited the number of IMUs participants were asked to wear during the three weeks of real-world LOB capture to reduce their level of inconvenience and thus to hopefully maximize adherence. Moreover, only sternum drop was investigated here. Although it is closely linked to two of the three kinematic requisites for successful trip recovery [20], future studies should include additional tripping kinematic measures to provide a more comprehensive comparison between settings. Fourth, the shoulder harness we elected to use to hold the sternum IMU did not provide a strong and direct connection between the IMU and sternum. As such, some relative movement between the IMU and the sternum may have occurred after some laboratory and real-world trips and thus contributed to variability within the sternum drop data. Future studies may benefit from an alternative approach while being mindful of participant comfort and convenience.

In conclusion, tripping kinematics after naturally occurring real-world trips were less severe than lab-induced trips, exhibited less kinematic variability than lab-induced trips, and showed no association between the two settings. While reducing gait speed and obstacle height in future laboratory studies may improve agreement with real-world trips, lowering the demands of balance recovery by doing to may not reveal contributing factors to falls as well as when demands are higher. Nevertheless, additional research appears warranted to clarify the relationship between lab-induced and real-world trips.

## Supporting information

S1 File Data file. This spreadsheet file includes the data analyzed in this paper.
(XLSX)
